# Anti-herpes simplex virus activities and mechanisms of marine derived compounds

**DOI:** 10.3389/fcimb.2023.1302096

**Published:** 2024-01-08

**Authors:** Cui Hao, Zhongqiu Xu, Can Xu, Ruyong Yao

**Affiliations:** ^1^ Medical Research Center, The Affiliated Hospital of Qingdao University, Qingdao, China; ^2^ Key Laboratory of Marine Drugs of Ministry of Education, Ocean University of China, Qingdao, China

**Keywords:** marine compound, herpes simplex virus, antiviral activity, molecular mechanism, therapeutic application

## Abstract

Herpes simplex virus (HSV) is the most widely prevalent herpes virus worldwide, and the herpetic encephalitis and genital herpes caused by HSV infection have caused serious harm to human health all over the world. Although many anti-HSV drugs such as nucleoside analogues have been ap-proved for clinical use during the past few decades, important issues, such as drug resistance, toxicity, and high cost of drugs, remain unresolved. Recently, the studies on the anti-HSV activities of marine natural products, such as marine polysaccharides, marine peptides and microbial secondary metabolites are attracting more and more attention all over the world. This review discusses the recent progress in research on the anti-HSV activities of these natural compounds obtained from marine organisms, relating to their structural features and the structure-activity relationships. In addition, the recent findings on the different anti-HSV mechanisms and molecular targets of marine compounds and their potential for therapeutic application will also be summarized in detail.

## 1 Introduction

Herpes simplex virus (HSV) is an enveloped double stranded DNA virus belonging to α herpesvirus subfamily of Herpesviridae, which has two serotypes: HSV-1 and HSV-2 (Sharma et al., 2016). Among them, HSV-2 mainly causes genital-skin and mucous membrane infection, while HSV-1 mainly causes oral, nasal, and ocular infection outside genitalia (James et al., 2020; Zhu and Viejo-Borbolla, 2021). Approximately 90% of the human population worldwide is sera-positive for HSV-1 or HSV-2 (Cohen et al., 2020). HSV can also produce a lifetime incubation period in neurons, and has the potential to cause more serious diseases, such as herpetic encephalitis, which may lead to death in severe cases (Gelfand, 2018; Kouyoumjian et al., 2018). Until now, there is no effective vaccine against HSV, and vaccine development is still a major challenge (Xu et al., 2019). Current treatments for HSV involve mainly nucleoside analogues, such as acyclovir (ACV) and its derivatives, such as valacyclovir, which mainly inhibit viral genome replication. Despite these successes, drug resistance and side effects remain unresolved issues in the fight against HSV infection (Piret and Boivin, 2011; Birkmann and Zimmermann, 2016). Therefore, it is critical to develop novel anti-HSV agents with high efficiency and low toxicity.

Marine organisms have produced a lot of structurally novel marine active molecules in special environments. Currently, 35000 natural products have been discovered from marine organisms, of which about 50% have been detected for biological activity (Laurienzo, 2010). These marine active molecules, as well as more undiscovered molecules, form a valuable “blue medicine bank”, which have always been the most fiercely competitive resource among countries around the world. In recent years, due to the continuous emergence of new viruses, there have been fewer and fewer drugs from terrestrial organisms, and the development of antiviral drugs has been slow (Wang et al., 2012). However, marine organisms have provided hope for the development of new antiviral drugs. Thus, the marine environment is considered an important source of active compounds targeting drug-resistant virus strains. Recently, researches on the anti-HSV activities of marine algae polysaccharides, marine peptides, and marine alkaloids have been continuously reported (Wang et al., 2012; Wang et al., 2014).

This review presents an overview of recent advances in research on the anti-HSV activities of marine derived compounds, relating to their structure features and structure–activity relationships. Moreover, this review will mainly focus on the sulfated marine polysaccharides in seaweed and bioactive molecules from invertebrate. Recent advances in the mechanisms of anti-HSV actions of marine compounds and their potential applications will also be discussed in detail.

## 2 Current clinical anti-HSV drugs and potential therapeutic strategies

Currently, the clinical drugs for treating herpes simplex virus infection are mainly acyclic nucleoside analogs (acyclovir, valacyclovir, etc.), acyclic nucleotide analogs (cidofovir, adefovir dipivoxil, etc.), and pyrophosphate inhibitors (sodium phosphoformate) (Coen and Schaffer, 2003). These three types of drugs all exert their anti-HSV actions by targeting the DNA replication process of HSV. Acyclovir (ACV) was first approved as an anti-herpes virus drug in 1977. When it functions, it is first phosphorylated by viral thymidine kinase to form monophosphate (ACVMP), and then catalyzed by cell kinase to form triphosphate (ACVTP). ACVTP then competitively acts as the substrate of DNA polymerase to inhibit the DNA synthesis of virus (Elion et al., 1977). Although ACV has high anti-HSV activity and good safety, it has some disadvantages such as low bioavailability and short half-life (Tilson et al., 1993). Cidofovir (CDV) is an acyclic nucleotide analog with a broad spectrum of anti-DNA virus activity (De Clercq et al., 1986). Its activation does not depend on viral kinase, and is also effective for HSV and VZV lacking thymidine kinase (De Clercq et al., 1987). Sodium phosphoformate is a pyrophosphate, which has antiviral activity against many viruses by inhibiting the pyrophosphate binding site of viral DNA polymerase, and also has activity against thymidine kinase deficient HSV and VZV (Wutzler, 1997).

**Figure 1:**
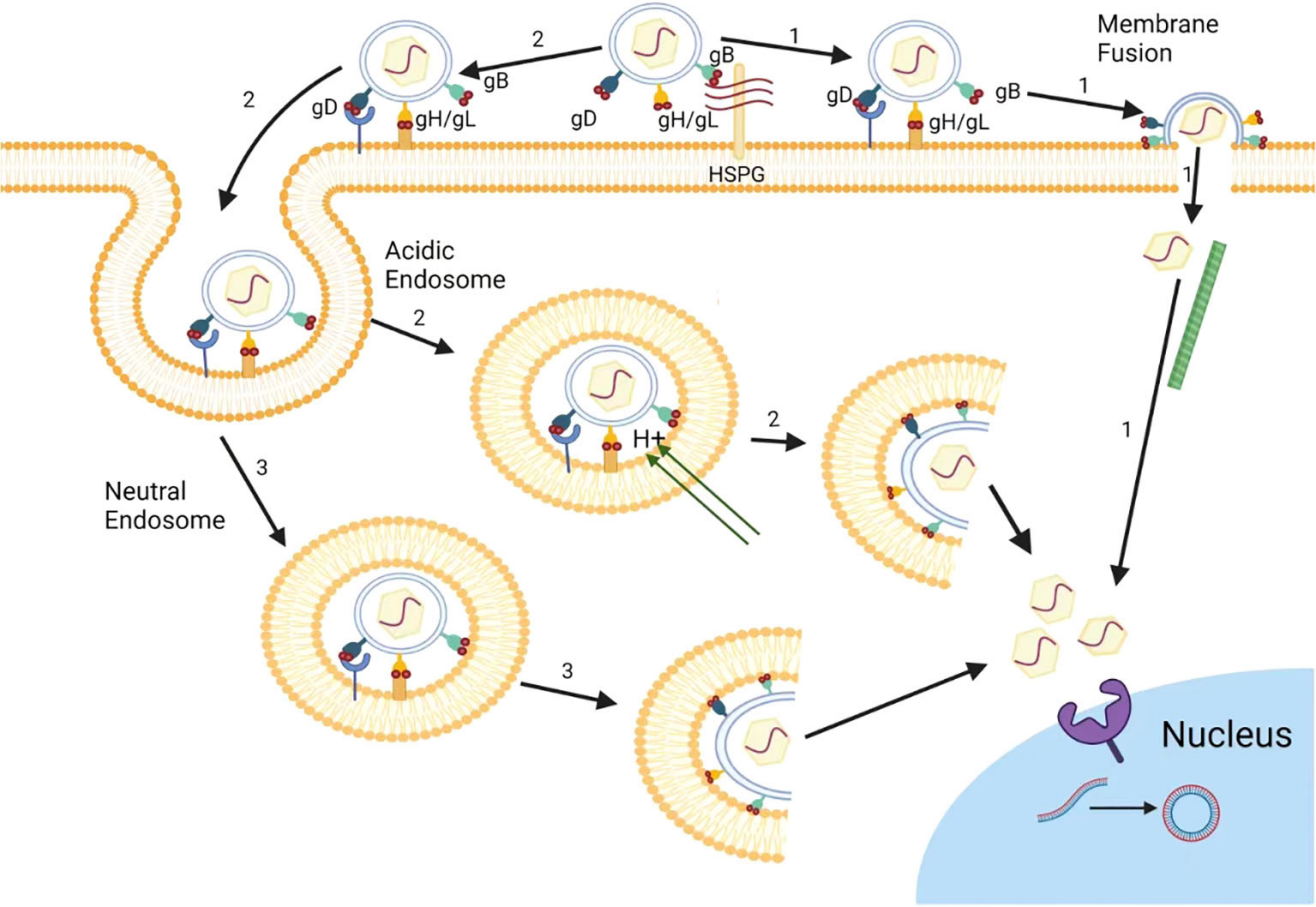
The entry pathways of herpes simplex virus. In Vero cells, HSV mainly releases its nucleocapsid into cell cytoplasm via inducing the direct fusion between HSV and cell membrane (1). In HeLa cells, besides cell surface fusion pathway (1), HSV can also release its nucleocapsid to cytoplasm through virus endocytosis and fusion with endosomal membrane (2) under acidic condition. In addition, HSV can also entry using endocytosis and endosome escape under neutral pH condition (3).

Furthermore, the efficient binding of a virus particle to its receptor on the cell surface is important for viral entry and subsequent replication steps in HSV life cycle (Hao et al., 2019). The envelope of HSV is a lipid bilayer containing 17 envelope proteins, including 12 envelope glycoproteins, namely gB, gC, gD, gE, gG, gD, gI, gJ, gK, gL, gM, and gN (Owen et al., 2015). Among them, four glycoproteins, gB, gD, gH, and gL, are necessary to induce cell membrane fusion, and are crucial for HSV to enter cells (**Figure 1**) (Turner et al., 1998). Thus, the prospect of drugs targeting HSV entry into host cells is promising, and many drugs have been proven to inhibit HSV entry by targeting receptors such as heparin sulfate (HS), or binding processes. Up to now, there are three common types of drug targets for the entry inhibitors of HSV: 1) Heparin sulfate (HS) analogue: It can competitively bind to the virus to inhibit HSV adsorption; 2) HS binding compound: It exert the anti-HSV effects by interacting with HS; 3) gD or gB targeted compound: It can directly interact with virus gD or gB proteins to block virus membrane fusion (**Figure 1**) (Campadelli-Fiume et al., 2012; Huang et al., 2022).

Despite the above success, the most anti-HSV drugs on the market generally have low oral bioavailability or short blood half-life, which cannot prevent patients from recurring symptoms, and long-term use of these drugs may lead to the emergence of drug-resistant strains (Tilson et al., 1993; Andrei and Snoeck, 2013). The researchers worldwide have invested a lot of effort in the development of vaccines for HSV, but so far, no vaccine has been validated or marketed to effectively prevent infection. Thus, it is important to develop novel anti-HSV agents with different mechanisms of action. Marine organisms as a vast source of compounds, provides the possibility for the search for new anti-HSV drugs.

## 3 Potential targets for the treatment of herpetic diseases

Owing to the emergence of drug resistance and side effects of nucleoside analogues, the need for novel anti-HSV drugs with divergent targets is highly sought after. Currently, novel targets for the development of new anti-HSV agents have been identified in the virus surface glycoproteins, viral VP5 protein, host heat shock proteins, and host kinases. Here, the main functional characteristics of novel targets for anti-HSV drug development are summarized in detail as follows.

### 3.1 Virus surface gB protein

HSV particle contains at least 12 envelope glycoproteins, which play different roles in virus adsorption, membrane fusion, and invasion processes. Among them, gB protein is mainly responsible for the membrane fusion with cell membrane or inner body membrane (Agelidis and Shukla, 2015). gB can bind to the heparan sulfate proteoglycan (HSPG) on cell surface to mediate the initial binding of HSV, and complete the membrane fusion and endocytosis process of HSV with the assistance of gD protein and gH/gL complex (Satoh et al., 2008; Arii et al., 2010; Suenaga et al., 2010; Weed and Nicola, 2017). gB can also interact with the gH/gL complex to induce membrane fusion and endocytosis processes, as well as mediating the budding and release of the viral nucleocapsid from the cell nuclear membrane (Farnsworth et al., 2007; Antoine et al., 2013; Agelidis and Shukla, 2015; Sathiyamoorthy et al., 2017). It was reported that the guanidine modified pyrimidine derivatives can interfere with the binding and entry processes of HSV by targeting gB protein (Wang W. et al., 2020). Besides that, the oligonucleotide molecule ODN5652 can inhibit the invasion of HSV into cells by inducing conformational changes in the gB protein (Shogan et al., 2006). Thus, the inhibitors of gB have the potential to be developed into novel anti-HSV agents in the future.

### 3.2 Virus surface gD protein

The gD protein exists on the surface of HSV virions in the form of homologous dimers, which contains approximately 394 amino acids (Arii and Kawaguchi, 2018). The receptors of gD on cell surface mainly include nectin-1, HVEM, and 3-OS HS, and different cell lines rely on different receptors to mediate the entry of HSV (Akhtar and Shukla, 2009). Besides that, the interaction between gD and gH/gL complex is also crucial for the membrane fusion process of HSV (Cairns et al., 2019). It was reported that a monoclonal antibody m27f targeting gD has good *in vitro* and *in vivo* anti-HSV activity, and can inhibit the membrane fusion process of HSV by binding to the pre-fusion domain of gD protein (Du et al., 2017). The sulfated gallic acid glucoside SPGG can significantly interfere with the adsorption and entry processes of HSV by targeting the gD protein (Majmudar et al., 2019). In addition, the RNA adapter with high gD affinity can effectively inhibit the invasion process of HSV-1 by binding to gD proteins (Gopinath et al., 2012).

### 3.3 Viral VP5 protein

Virus protein 5 (VP5), also known as ICP5, is the main structural component of the virus capsid, and can form the capsomere of HSV together with three other capsid proteins, VP19C, VP23, and VP26 (Heming et al., 2017). The assembly of viral capsids is mainly driven by the interactions between VP5 and other virus proteins such as VP22a, UL25, VP26, and ICP35 (Walters et al., 2003; Huet et al., 2016). The seven hydrophobic amino acids at the N-terminus of VP5 are crucial for VP5 binding to scaffold proteins and ultimately forming a closed icosahedral shell (Musarrat et al., 2021). In addition, VP5 can also mediate the transport of the viral shell to the nucleus by interacting with the dynactin cofactor (Jin et al., 2014). Jin et al. found that the siRNAs targeting the expression of VP5 and VP23 can significantly inhibit HSV proliferation *in vitro* (Mues et al., 2015). In addition, the small molecule inhibitor Dynasore can interfere with the co-localization of VP5 and dynamin, thereby inhibiting the capsid transport process of HSV (Jin et al., 2014).

### 3.4 Host heat shock protein 90

Heat shock protein 90 (Hsp90) is a highly conserved molecular chaperone that plays essential roles in constitutive cell signaling and adaptive responses to stress, such as microbial infection (Crevel et al., 2001; Maloney and Workman, 2002; Ratzke et al., 2010). Hsp90 has been shown to be important for many different viruses that require chaperone functions for viral protein folding, replication, transport, and assembly (Geller et al., 2012). It was reported that the Hsp90 inhibitors can block HSV-1 nuclear egress and assembly in Vero cells (Li et al., 2018), and Hsp90 is essential for the correct localization of HSV-1 DNA polymerase to the nucleus (Burch and Weller, 2005). Besides that, Hsp90 also promotes nuclear transport of HSV-1 capsid proteins by interacting with acetylated tubulin (Zhong et al., 2014; Li et al., 2019). In addition, the Hsp90 inhibitors can also inhibit the entry of HSV-1 into neuron cells by regulating cofilin-mediated F-actin reorganization (Song et al., 2022).

### 3.5 Cyclin-dependent kinases

Cyclin-dependent kinases (CDKs) regulate the cell division cycle, apoptosis, transcription and differentiation in addition to functions in the nervous system (Knockaert et al., 2002). It was found that CDK1, 2, or 7 is required for HSV replication in nonneuronal cells, while CDK2 is required for HSV-1 reactivation in neurons (Schang et al., 1998; Schang, 2004). The CDK inhibitors can inhibit HSV-1 replication by interfering with the transcription of viral IE, E, and L genes (Schang et al., 1999). BMS-265246 (BMS), a CDK 1/2 inhibitor, may limit HSV-1 multiplication through interfering with multiple steps in HSV-1 replication (Jiang et al., 2023). Besides, CDK inhibitors can also inhibit the replication of many other viruses, including HCMV (Bresnahan et al., 1997), HSV-1 (Schang et al., 2000), VZV (Moffat et al., 2004), and HIV-1 (Guendel et al., 2010), suggesting that CDKs may be prospective antiviral targets.

## 4 Anti-HSV effects of natural compounds obtained from marine organisms

Antiviral active substances from the ocean mainly exist in marine animals and plants such as sponges, ascidians, seaweeds, and marine microorganism associated with them. The common types of anti-HSV compounds are mainly polysaccharides, terpenoids, nucleosides, alkaloids and peptides. The first approved marine anti-HSV drug vidarabine is a nucleoside compound derived from Sarcandra angustifolia, which can be used to treat herpetic encephalitis and herpes simplex keratitis (Sadowski et al., 2021). The main structural characteristics and anti-HSV effects of marine derived active compounds are summarized in detail as follows.

### 4.1 Anti-HSV compounds derived from seaweeds

Seaweed is a source of natural products with many biological applications, including substances that inhibit viral infection or replication. Algae are mainly divided into two types, namely macroalgae and microalgae. Macroalgae occupy coastal areas, including red algae, brown algae, and green algae, while microalgae live in deep-sea water columns, sediments, and coastal habitats, including diatoms, dinoflagellates, brown flagellates, and blue-green algae (Gutiérrez-Rodríguez et al., 2018). Marine algae produce various metabolites and have been recognized as the promising sources for discovering anti-HSV compounds.

#### 4.1.1 Red algae derived compounds

Marine sulfated polysaccharides derived from red algae often possess marked anti-HSV activities, mainly through inhibiting virus attachment to cell surfaces (Pliego-Cortés et al., 2022). The sulfation content is the key factor which influences the anti-HSV effects of other red algae derived polysaccharides. Carrageenan, a red alga derived sulfated polysaccharide, possesses different inhibition effects on different viruses, including HSV (Carlucci et al., 2004; Talarico and Damonte, 2007; Grassauer et al., 2008; Leibbrandt et al., 2010; Jiao et al., 2011; Chiu et al., 2012). Natalia et al. found that carrageenans effectively inhibited HSV infection mainly through binding to virus glycoprotein gD to prevent virus-cell interactions (Krylova et al., 2022). Carrageenan polysaccharides exhibited antiviral activities against both HSV-1 and HSV-2 *in vitro*, and also showed significant inhibition against HSV-2 vaginal infection in mouse models (Talarico et al., 2004). Besides that, the polyelectrolyte complex (PEC) composed of carrageenan and chitosan were found to be able to effectively inhibit the early stages of HSV infection, superior to the effect of carrageenan or chitosan alone (Davydova et al., 2023). Thus, the combination of carrageenan with other polysaccharides may become an important strategy for the development of anti-HSV drugs (Bouhlal et al., 2011; Lomartire and Gonçalves, 2022; Davydova et al., 2023).

**Figure 2:**
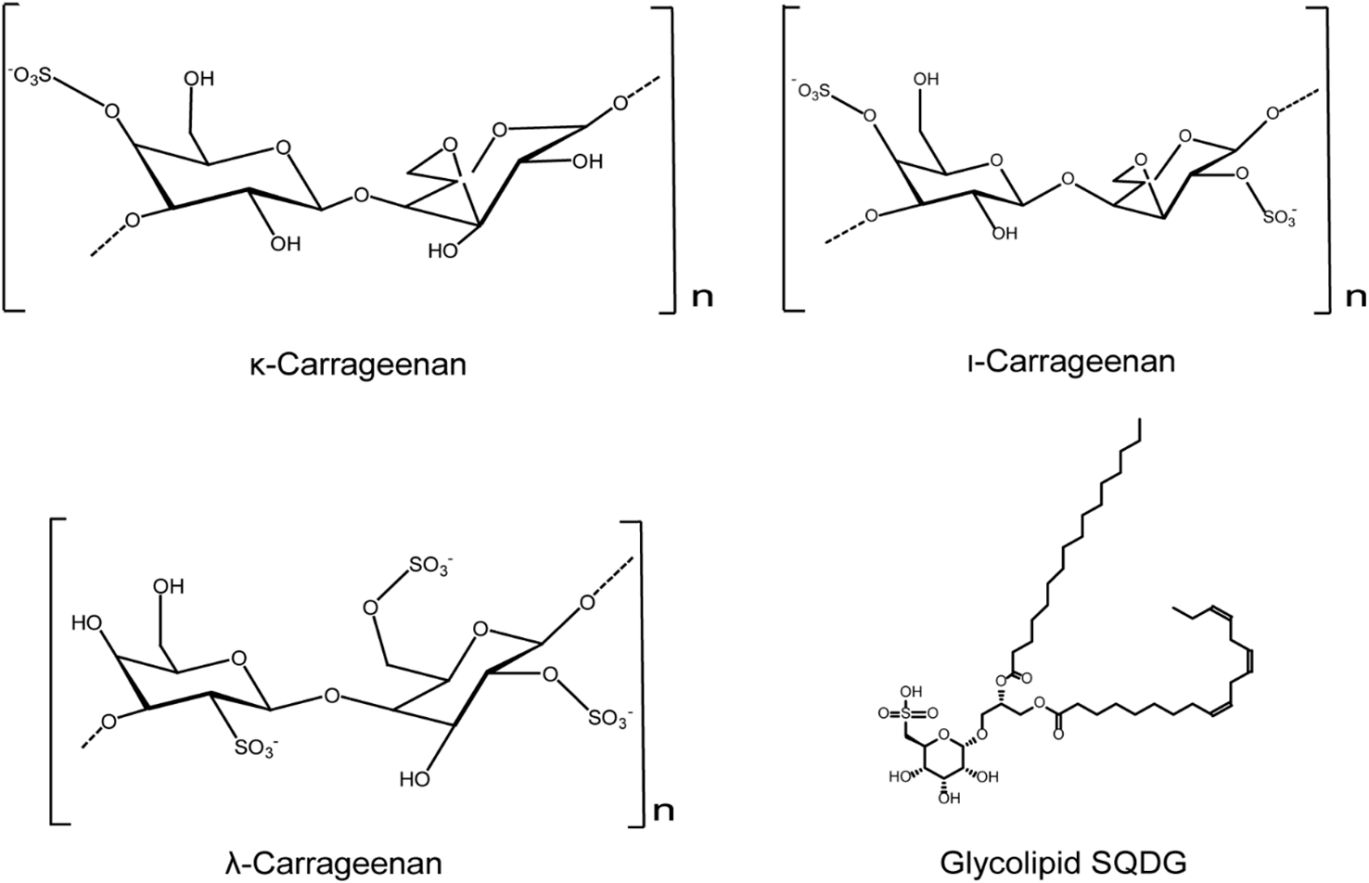
Chemical structures of red algae derived compounds with anti-HSV effects. κ-carrageenan and ι-carrageenan may directly bind to HSV to inactivate the HSV virions. λ-carrageenan may bind to HSV receptors to block HSV adsorption process. Compound SQDG exhibits strong antiviral activity against both HSV-1 and HSV-2 *in vitro* (see the text for all details).

Furthermore, griffithsin (GRFT), a mannose binding lectin extracted from red algae, was also reported to exert anti-HSV-2 activities mainly through inhibiting the secretion and transmission of HSV-2 after infection (Barton et al., 2016). Lauro et al. found that the glycolipid SQDG exhibited strong antiviral activity against both HSV-1 and HSV-2, with IC_50_ values of less than 50 µg/mL (De Souza et al., 2012). In summary, red algae derived compounds such as the sulfated polysaccharides and glycolipids possess marked anti-HSV activities, and their antiviral actions may be related to the optimal molecular weights and sulfation levels (**Figure 2**).

#### 4.1.2 Green algae derived compounds

**Figure 3:**
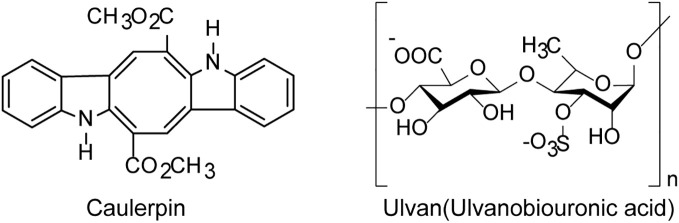
The chemical structures of anti-HSV compounds derived from green algae. The indole alkaloid Caulerpin may be used as a novel anti-HSV agent to inhibit some stages of HSV replication cycle. The sulfated polysaccharide Ulvan shows a high anti-herpetic effect, especially on HSV-1 (see the text for all details).

Green algae derived polysaccharides such as ulvans was reported to have inhibition effects on various viruses including EV71 and HSV (Tziveleka et al., 2019) (**Figure 3**). Lopes et al. found that the sulfated polysaccharide SU1F1 from green algae had a high anti-herpetic effect, especially on HSV-1, and the higher the degree of sulfonation of this molecule, the better its anti-HSV efficacy (Lopes et al., 2017). Besides, the green algae derived small molecules such as alkaloids also possess anti-HSV activities *in vitro*. The indole alkaloid Caulerpin derived from the green alga *Caulerpa Lamouroux*, had anti-HSV effects *in vitro* with the IC_50_ value of 1.29 μg/mL, superior to the effect of acyclovir (ACV), and Caulerpin may be used as a novel anti-HSV agent to inhibit some stages of viral replication cycle (Macedo et al., 2012). In addition, the ethanolic extract of green algae *Spirogyra* spp. such as terpenoids, alkaloids and essential oils presented high inhibition on HSV-1 infection with the IC_50_ value of 2.17 μg/ml (Deethae et al., 2018).

#### 4.1.3 Brown algae derived compounds

Brown algae produce different polysaccharides, including alginates, fucoidans and laminarans, which possess different therapeutic properties and relatively low toxicity (Hayashi et al., 2008; Wang et al., 2008; Laurienzo, 2010; Lee et al., 2012; Takeda et al., 2012; Khajouei et al., 2018). The fucoidans derived from brown algae *Nizamuddinia zanardini* showed strong anti-HSV-2 activity, and may mainly inhibit the early stages of HSV life cycle (Baba et al., 1988; Mandal et al., 2007; Alboofetileh et al., 2019). Sun and co-workers extracted fucoidans SHAP-1 and SHAP-2 from brown algae *Sargassum henslowianum*, and found that these two fucoidans exhibited good antiviral activities against both HSV-1 and HSV-2, with IC_50_ values less than 0.9μg/ml (Sun et al., 2020). Moreover, many other brown algae polysaccharides have also been identified to have good anti-HSV activities, and these polysaccharides may possibly affect the entry process of HSV-1 (Bandyopadhyay et al., 2011; Somasundaram et al., 2016; Sanjeewa et al., 2018). Estefania et al. found the water extracts from two brown algae had marked antiviral activities against both HSV-1 and HSV-2, mainly through blocking some replication events of HSV after virus entry (Castillo et al., 2020).

**Figure 4:**
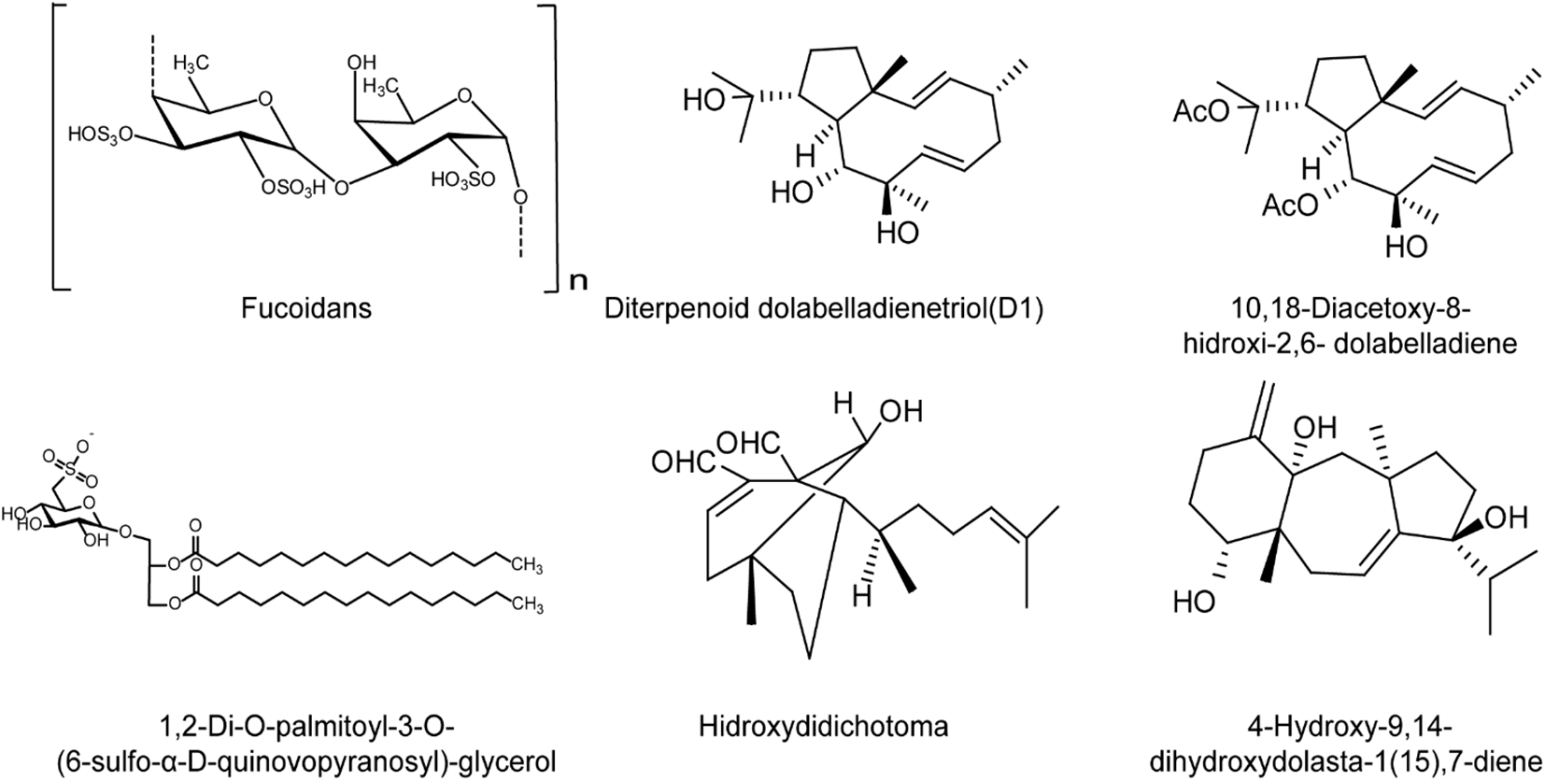
The chemical structures of brown algae derived compounds with anti-HSV effects. Fucoidans may mainly inhibit the early stages of HSV life cycle *in vitro*, and inhibit HSV infection in mice mainly through enhancement of host immune functions to block viral replication. The diterpenoid dolabelladienetriol (D1) inhibits HSV-1 infection both *in vitro* and *in vivo* in a dose-dependent manner. The diterpenoid compound hidroxydidichotoma also inhibits the replication of HSV-1 *in vitro* in dose- and MOI-dependent manner. The glycolipid SQDG shows strong anti-HSV-1 and HSV-2 activities *in vitro*. The diterpenes 10,18-trihydroxy-2,6-dolabelladiene and dihydroxydolasta-1,7-diene may inhibit initial events during HSV-1 replication rather than impair virus entry (see the text for all details).

Furthermore, some terpenoid components isolated from brown algae also showed anti-HSV activity. The diterpenoid dolabelladienetriol (D1) isolated from the Brazilian brown algae *Dictyota pfafii* inhibited HSV-1 infection both *in vitro* and *in vivo* in a dose-dependent manner, similar to the effect of acyclovir (ACV) (Garrido et al., 2017). Besides, the glycolipid SQDG isolated from the brown algae *Sargassum vulgare* exhibited strong anti-HSV-1 and HSV-2 activities *in vitro* (Plouguerné et al., 2013). The diterpenes 10,18-trihydroxy-2,6-dolabelladiene and dihydroxydolasta-1,7-diene, isolated from the brown algae may inhibit initial events during HSV-1 replication rather than impair virus adsorption and penetration (Abrantes et al., 2010). In addition, the diterpenoid compound hidroxydidichotoma can also inhibit the replication of HSV-1 *in vitro* in significant dose- and MOI-dependent manner (Abrantes et al., 2010), suggesting that the terpenoids from brown algae are also expected to be used in the development of anti-HSV drugs in the future (**Figure 4**).

#### 4.1.4 Microalgae derived compounds

Nowadays, lots of valuable compounds have been isolated from microalgae, including lipids, pigments, peptides, polysaccharides, minerals or vitamins, many of which have shown significant antiviral activities, including anti-HSV effects. Srivastava et al. reported that the microalgae polysaccharide Ca-SP can inhibit the replication of several envelope viruses, including HSV-1, human cytomegalovirus, influenza A virus and HIV-1 (Srivastava et al., 2022; Chen et al., 2023). Further studies indicated that treatment of Ca-SP two hours before infection has a strong inhibitory effect against HSV-1, indicating that it mainly blocks virus adsorption and entry into host cells (Srivastava et al., 2022). Moreover, Esmail et al. discovered that a new lectin from the blue-green algae significantly inhibited the plaque formation in HSV-1 infected Vero cells, and it may block the initial step of HSV infection via directly acting on HSV virions (El-Fakharany et al., 2020). Recently, it has been reported that compound cyanovirin-N, can block the infection of HSV-1 through inhibiting the membrane fusion process mediated by virus glycoproteins (Tiwari et al., 2009).

### 4.2 Anti-HSV agents from marine microbes

**Figure 5:**
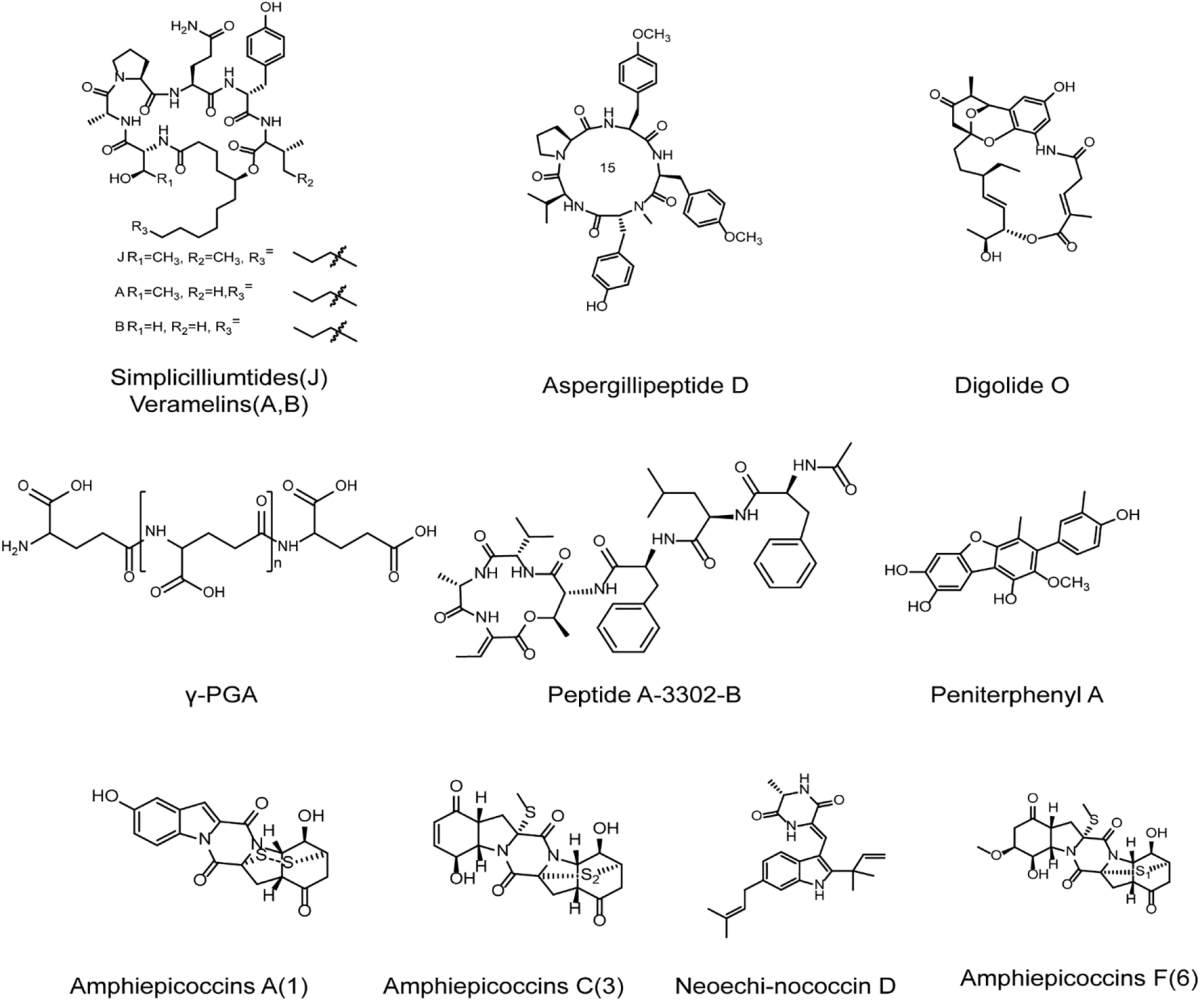
The chemical structures of anti-HSV compounds from marine microbes. γ-PGA may block the early stages of HSV replication so as to hinder HSV-1 infection. Peptide A-3302-B may largely inhibit the late events of HSV-2 infection. Digolide O exhibits marked anti-HSV-1 activity with low cytotoxicity. Peniterphenyl A may interfere with HSV adsorption and entry processes through direct interaction with viral gD protein. Amphicoccins A, C, and F exhibit marked anti-HSV-2 activities. Neoechinococcin D shows almost 100% inhibition on HSV-1. The cyclic peptides Simpliciliumtides J and Aspergillipeptide D exhibit marked anti-HSV activities and Aspergillipeptide D may exert anti-HSV actions trough inhibit the expression and transport of virus gB protein. Peptide Halovirs A can directly inactivate HSV-1 to exert marked anti-HSV activity (see the text for all details).

So far, marine microorganism is considered as a relatively underestimated source of bioactive compounds, and can provide compounds with new structures and antiviral activities (Adnan et al., 2018; Stien, 2020). The anti-HSV research of marine microorganism has received little attention, but marine bacteria and fungi are still very promising sources of anti-HSV drugs (**Figure 5**).

#### 4.2.1 Compounds derived from marine bacteria

Compounds derived from marine bacteria can exert anti-HSV effects by affecting the early or late stages of HSV infection. The γ-Poly (glutamic acid) (γ-PGA) produced by marine heat-resistant *Bacillus Horneckiae* was reported to be able to block the early stages of HSV replication so as to hinder HSV-1 infection, and regulate the expression of TNF-α and IL-1β (Marino-Merlo et al., 2017). Sureram et al. reported that the peptide A-3302-B can largely inhibit the late events of HSV-2 infection, different from the anti-HSV mechanism of γ-PGA (Sureram et al., 2022). Moreover, it was reported that the amphotericin derivative digolide O isolated from marine Streptomyces exhibited marked anti-HSV-1 activity, and showed low cytotoxicity to Vero cells (Nong et al., 2020). Furthermore, the three extracellular polysaccharides EPS1-B3-15, EPS1-T14, and EPS2 isolated from marine bacteria all significantly inhibited HSV-2 replication in peripheral blood mononuclear cells (PBMCs), suggesting that the marine bacteria derived extracellular polysaccharides can be used for the therapy of HSV infection (Arena et al., 2009; Gugliandolo et al., 2014; Spanò and Arena, 2016).

#### 4.2.2 Compounds from marine fungi

The small molecular compounds derived from marine fungi also have good anti-HSV activities. Chen et al. reported that compound Peniterphenyl A isolated from deep-sea penicillium SCSIO41030 may be able to interfere with HSV adsorption and entry processes through direct interaction with viral gD protein, different from the mechanisms of nucleoside analogs such as acyclovir (Chen et al., 2021). In addition, it was reported that the inhibition rate of HSV-1 by emodin A and neoechinococcin D isolated from the fungi in the sponge was almost 100% (Bovio et al., 2019). Recently, some peptides isolated from marine fungi were reported to have antiviral activities, and have been applied in various biomedical fields. It was reported that the cyclic peptides simpliciliumtides J and Aspergillipeptide D isolated from marine fungus exhibited marked antiviral activities against different HSV strains such as HSV-1/F (F strain), HSV-1/106 (ACV resistant strain), and HSV-1/153 (ACV re-sistant strain) (Liang et al., 2017; Wang Z. et al., 2020). Aspergillipeptide D can reduce the expression levels of viral glycoprotein gB, and inhibit the localization of gB in Golgi apparatus and endoplasmic reticulum, thus playing an anti-HSV role *in vitro* (Wang Z. et al., 2020). Besides, another peptide Halovirs A isolated from *Scytalidium fungi* can directly inactivate HSV-1 to exert marked anti-HSV activity (Rowley et al., 2003).

### 4.3 Bioactive compounds from marine invertebrates

**Figure 6:**
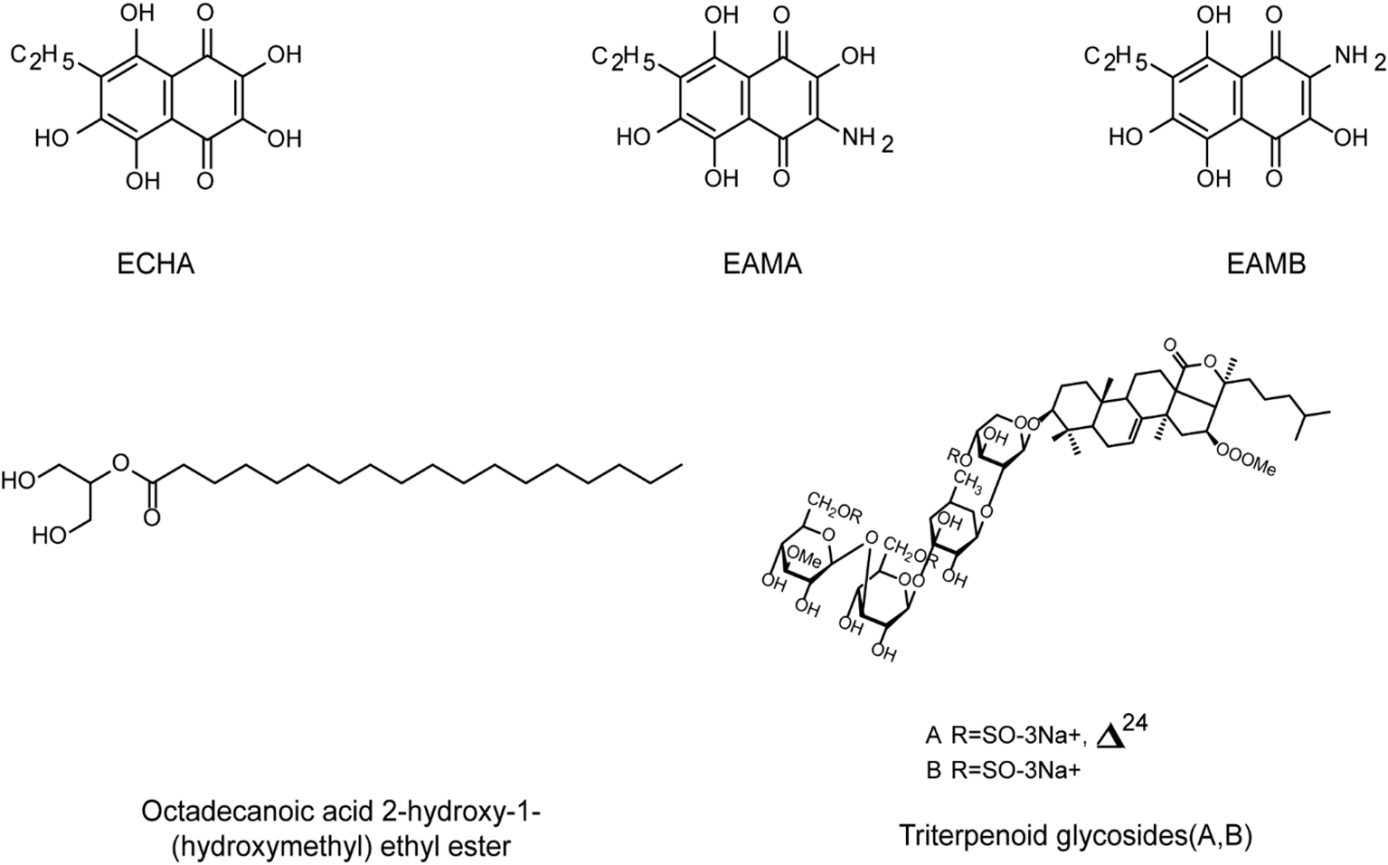
The chemical structures of anti-HSV compounds from marine invertebrates. Echinochrome ECHA and its analogues EAMA and EAMB can significantly reduce the plaque formation of HSV-1 in Vero cells, with low toxicity. EAMA and EAMB may directly bind to virus gD protein to prevent virus adsorption. Triterpenoid glycosides A and B show antiviral activities against HSV-1 below 10 µg/mL. Octadecanoic acid ether ester may interact with the binding domain of the virus and cell receptors, leading to interference with virus adsorption (see the text for all details).

Different from the immune defense system of marine vertebrates, the common in-vertebrates including sponges, tunicates, echinoderms, and mollusks only have an innate immune system and therefore often produce some secondary metabolites to assist them in resisting the threat of exogenous pathogenic organisms (Xiao et al., 2022). Thus, the secondary metabolites of marine invertebrates often possess antibacterial and antiviral activities, which can be used to develop novel anti-HSV drugs (**Figure 6**).

Sponges can produce a large number of anti-tumors, antiviral, anti-inflammatory, and other potentially therapeutic bioactive molecules, which can become raw materials for the development of antiviral drugs. Lhulier et al. reported that the chloroalkane diterpenes extracted from the sponge *Rasailia bouryesnaultae* showed a replication inhibition rate of more than 50% for HSV-1 (KOS strain) and over 70% for HSV-1 (29R strain), respectively (Lhullier et al., 2019). Besides that, it was reported that three types of cyclic peptide didemnins (A-C) isolated from *tunicates* can inhibit the reproduction of various RNA or DNA virus *in vitro*, with the IC_50_ values less than 0.1 μM (Rangel et al., 2017). Zhou et al. found that the ethanol extract of *tunicates* showed inhibitory effect on HSV-2 replication, and further proved that its target was the DNA polymerase UL30 gene of HSV-2 (ZP et al., 2015).

Moreover, due to the diverse biological activities of their secondary metabolites, echinoderms have become the great potential sources of antiviral drugs. Mishchenko et al. reported that the sea urchin derived echinochrome analogues EAMA and EAMB can significantly reduce the plaque formation of HSV-1 in Vero cells, and they may directly bind to virus gD protein to compete for the binding sites between the protein and cell receptors, thereby preventing virus adsorption on cells (Mishchenko et al., 2020). In addition, two new triterpenoid glycosides A and B isolated from sea cucumber showed antiviral activities against HSV-1 at concentrations below 10 ug/mL (Maier et al., 2001). Moreover, the main components of the ethanol extract of sea cucumber are Octadecanoic acid ether ester and cetoxylide, which showed low cytotoxicity to Vero cells (Keshavarz et al., 2021). Further studies showed that these two components may interact with the binding domain of the virus and cell receptors, leading to interference with the attachment of HSV to its cell receptors (Farshadpour et al., 2014).

Furthermore, molluscs lack an adaptive immune system and play an antiviral role mainly by secreting effectors with immune regulation function, including antimicrobial peptides, hemocyanin and hemolymph proteins (Xiao et al., 2022). The abalone hemocyanin extracted from abalone *Haliotis Rubra* had a dose-dependent inhibitory effect on HSV-1 infection *in vitro*, and it may selectively bind to the surface glycoproteins gB, gD, and gC of the virus, thereby inhibiting the adsorption and entry of HSV (Farshadpour et al., 2014). However, haemocyanin had no effect on the late stages of HSV-1 life cycle and did not directly bind to the cell receptors of HSV (Farshadpour et al., 2014). Taken together, the secondary metabolites from marine invertebrates merit further investigations to be developed into new anti-HSV drugs in the future (**Figure 6**).

## 5 Anti-HSV mechanisms and molecular targets of marine compounds

**Figure 7:**
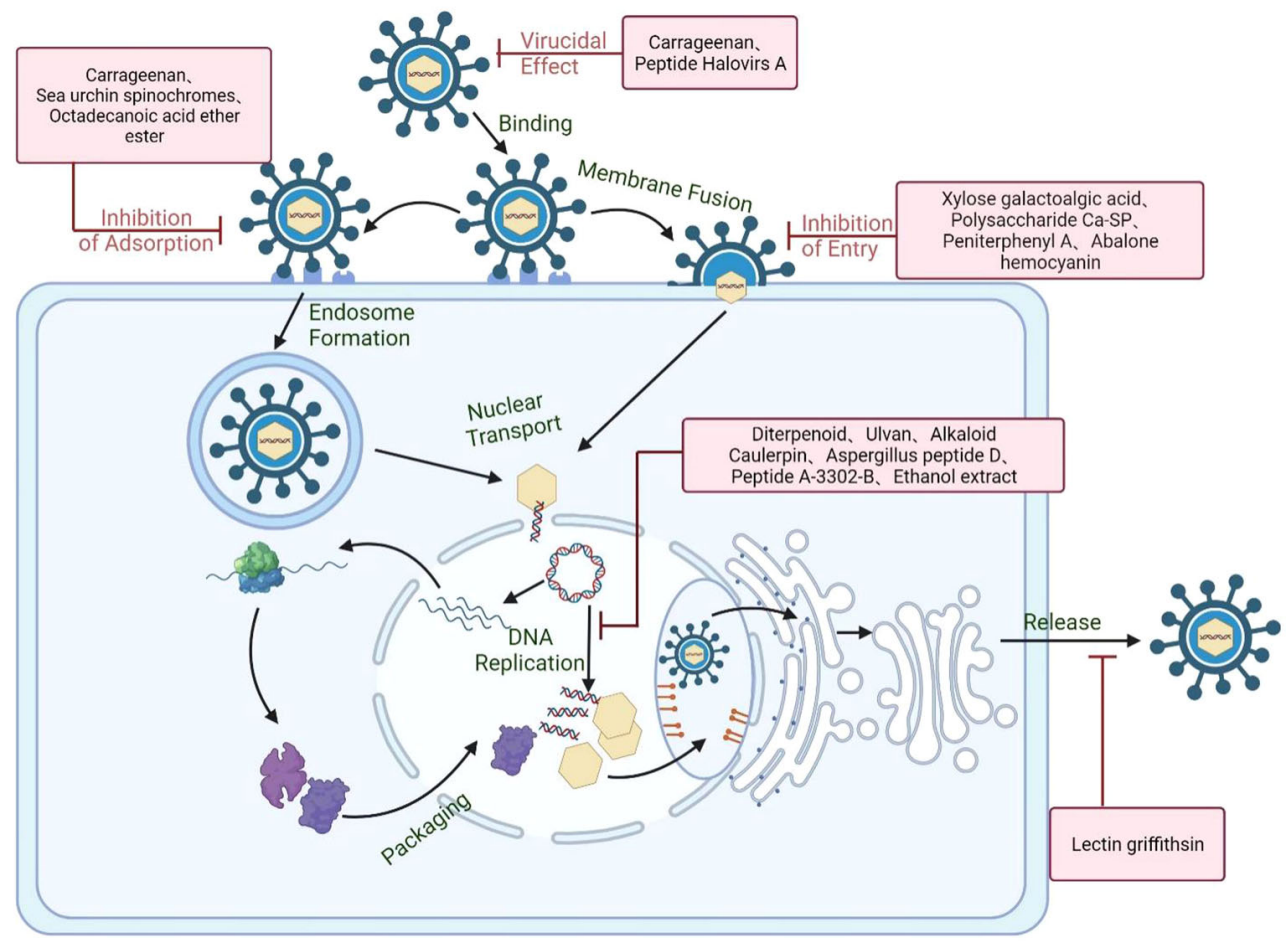
Schematic diagram of the anti-HSV mechanisms of marine compounds. Marine compounds can directly inactivate the HSV particle and block virus initial attachment to cell surface receptors such as HSPG or other specific receptor. Marine compounds may also block the genome release through interfering with virus endocytosis, membrane fusion, and nucleocapsid transport. Besides, some marine compounds can also inhibit virus transcription and replication processes as well as enhancing the antiviral immune system. Created with BioRender.com.

Marine derived compounds such as the algae polysaccharides and the secondary metabolites of marine organisms can either block the infection of HSV through interfering with viral life cycle or enhance the host antiviral immune responses to improve the viral clearance (**Figure 7**) (Damonte et al., 2004; Wang et al., 2012). Like other viruses, there are five main stages in the life cycle of HSV: viral adsorption, viral entry, uncoating of capsids, replication, viral assembly and release (Huang et al., 2022). The anti-HSV mechanisms and universal targets of marine compounds will be discussed in detail (**Table 1**).

**Table 1:** Anti-HSV mechanisms of selected marine compounds.

Marine Organisms	Specific Compounds	Mechanisms of Action	References
Red Algae	Lectin griffithsin	Inhibition of transmission	(Barton et al., 2016)
	Carrageenan	Virucidal effect	(Carlucci et al., 1999a; Carlucci et al., 2002; Harden et al., 2009)
	Carrageenan	Inhibition of adsorption	(Carlucci et al., 1997; Carlucci et al., 1999b; Mazumder et al., 2002)
Brown Algae	Fucoidan	Enhancing immune system	(Hayashi et al., 2008)
	Diterpenoid	Inhibition of replication	(Plouguerné et al., 2013; Garrido et al., 2017)
Green Algae	Ulvan	Inhibition of replication	(Tziveleka et al., 2019)
	Alkaloid Caulerpin	Inhibition of replication	(Macedo et al., 2012)
Microalgae	Polysaccharide Ca-SP	Inhibition of entry	(Srivastava et al., 2022; Chen et al., 2023)
Marine fungus	Peniterphenyl A	Inhibition of entry	(Chen et al., 2021)
	Aspergillus peptide D	Inhibition of replication	(Liang et al., 2017; Wang Z. et al., 2020)
	Peptide Halovirs A	Virucidal effect	(Rowley et al., 2003)
Marine bacterium	γ-Poly (glutamic acid)	Enhancing immune system	(Marino-Merlo et al., 2017)
	Peptide A-3302-B	Inhibition of replication	(Sureram et al., 2022)
Tunicates	Ethanol extract	Inhibition of replication	(ZP et al., 2015)
Echinoderms	Sea urchin spinochromes	Inhibition of adsorption	(Mishchenko et al., 2020)
	Octadecanoic acid ether ester (Sea cucumber)	Inhibition of adsorption	(Keshavarz et al., 2021)
Molluscs	Abalone hemocyanin	Inhibition of entry	(Farshadpour et al., 2014)

### 5.1 Direct virucidal action

Many marine compounds can directly act on the surface proteins or lipid membranes of HSV to disrupt the structural integrity of virions, thereby rendering HSV incapable of infection. Carrageenan is a sulfated polysaccharide with negative charges, which may inhibit viral infection by directly acting on the surface of HSV through electrostatic interaction. Carrageenan was reported to be able to firmly bind to HSV to change the structure of HSV glycoprotein gB and gC, thus leading to the inactivation of HSV virions (Carlucci et al., 1999a; Carlucci et al., 2002; Harden et al., 2009). The virucidal effects of carrageenan may be due to the formation of virion–carrageenan complex so as to block the sites on the viral envelope required for virus adsorption to host cells (Neyts et al., 1992). Moreover, some marine derived small molecular compounds can also directly inactivate the HSV via changing the integrity of virus envelope. It was reported that the marine peptide Halovirs A isolated from Scytalidium fungi can directly bind to HSV particles to inactivate HSV-1 virions (Rowley et al., 2003). Taken together, some marine compounds may have direct virucidal actions on HSV virions so as to block the subsequent infection of HSV (Antoine et al., 2013).

### 5.2 Inhibition of viral adsorption

The adsorption process is the first infection step of HSV life cycle, and cell surface glycosaminoglycan (GAG) such as heparin is often the initial receptor of human herpes virus HSV-1, HSV-2, and bovine herpes virus (Okazaki et al., 1991; Kari and Gehrz, 1992). In general, marine compounds may affect the physiological status of viral entry receptors through two main ways: 1) Direct interaction with receptors to block virus adsorption; 2) Regulating intracellular signaling to affect receptor mediated endocytosis (Okazaki et al., 1991; Kari and Gehrz, 1992; Mazumder et al., 2002). Heparinoid polysaccharides such as carrageenans can block viral adsorption by inhibiting the interaction between HSV and the initial receptors on cell surface. Mazumder et al. found that the high-molecular-weight carrageenan exhibited anti-HSV activities mainly through blocking the initial viral adsorption to the host cells (Mazumder et al., 2002). Besides, λ-carrageenan from *Gigartina skottsbergii* can firmly bind to HSV receptors in order to block the attachment of HSV to the host cell surfaces (Carlucci et al., 1997; Carlucci et al., 1999b). Moreover, spinochromes derived from sea urchins were reported to be able to directly bind to HSV gD protein to compete for the binding site of this protein with cell receptors (3-OS HS and Nectin-1), thereby preventing virus adsorption on cell surface (Mishchenko et al., 2020). In addition, the sea cucumber derived Octadecanoic acid ether ester exhibited the anti-HSV action mainly through interference with the attachment of HSV to host cell receptors (Keshavarz et al., 2021).

### 5.3 Inhibition of virus entry process

The common entry process of viruses usually involves the membrane fusion, endocytic uptake, vesicular transport, delivery to endosomes and virus uncoating (Mercer et al., 2010). The uncoating of HSV capsids usually occurs after viral endocytosis, as well as in the same time of viral fusion with cell membrane. The sulfated polysaccharides derived from brown algae such as alginic acid possessed good anti-HSV effects, and their antiviral actions may be due to the inhibition on the entry process of HSV (Bandyopadhyay et al., 2011). Peniterphenyl A isolated from Penicillium SCSIO41030 can inhibit HSV infection mainly through binding to gD protein to block virus membrane fusion process, different from the mechanisms of acyclovir (Chen et al., 2021). Talaei et al. found that the abalone hemocyanin can selectively bind to the glycoproteins gD, gB and gC on the envelop of the virus, thus inhibiting the entry process rather than the late events of HSV infection (Farshadpour et al., 2014). Thus, marine derived compounds can also inhibit HSV infection through blocking membrane fusion and the subsequent endocytosis and genome release processes.

### 5.4 Inhibition of virus replication

Like the current approved anti-HSV drugs such as acyclovir, some marine compounds can also inhibit viral transcription and replication through interfering with viral replication enzymes or other intracellular targets. Estefania et al. found that the water extracts of brown algae exerted anti-HSV activity mainly through blocking the replication cycle of HSV in the steps after virus entry (Castillo et al., 2020). Carrageean may also block some replication events subsequent to HSV internalization but prior to the onset of late viral gene expression (González et al., 1987). Moreover, the indole alkaloid Caulerpin isolated from green alga may exert its anti-HSV effects through inhibiting the replication cycle of the virus α and β stages (Macedo et al., 2012). In addition, the ethanol extract of Styela plicata showed inhibitory effect on HSV-2 replication mainly through targeting the DNA polymerase UL30 gene of HSV-2 (ZP et al., 2015). Besides that, the cyclic aspergillus peptide D can significantly inhibit HSV-1 replication through reducing the expression levels of viral gB protein, and decreasing the localization of gB in Golgi apparatus and endoplasmic reticulum (Wang Z. et al., 2020).

### 5.5 Enhancement of host immune responses

Virus infection can often induce the host antiviral immune responses, of which the type I and type II interferon system is the host’s first line of defense against viral infections (Wang et al., 2012). Thus, if marine compounds can activate immunocytes or enhance the generation of antiviral immune factors, they may also inhibit HSV replication or accelerate the clearance of HSV. The sulfated polysaccharide fucoidan isolated from the brown alga was reported to possess anti-HSV effects both *in vitro* and *in vivo*, and may inhibit HSV infection in mice mainly through enhancement of host immune functions to block viral replication (Hayashi et al., 2008). Besides, carrageenan polysaccharides can also markedly improve the activity of NK cells and increase the proliferation rate of lymphocytes (Zhou et al., 2004). Furthermore, some cellular signaling pathways such as the nuclear factor κB (NF-κB) pathway play important roles in the activation of innate immune responses through inducing the gene expression of antiviral factors. However, HSV have developed multiple ways to inhibit the activation of NF-κB so as to escape the host antiviral response (Li et al., 2023). Thus, enhancing the activation of NF-κB pathway and related cytokines may be able to indirectly inhibit HSV infection.γ-Poly (glutamic acid) (γ-PGA) produced by marine Bacillus can not only block the early stages of HSV replication but also exhibit the immune regulation actions by enhancing the expression of TNF-α and IL-1β, thus inhibiting the replication of HSV (Marino-Merlo et al., 2017). Therefore, marine compounds can also accelerate HSV clearance through enhancing the antiviral immune system.

## 6 Constraints on the development of anti-HSV marine drugs

Recently, researches on the anti-HSV activities of marine polysaccharides, peptides and alkaloids have been continuously reported. Although most marine sulfated polysaccharides have good anti-HSV effects, they have not been developed as novel anti-HSV drugs due to their difficulty in absorption and low oral availability. At present, the extraction, purification, and efficacy evaluation of polysaccharides have become relatively mature. However, research on its pharmacokinetics, especially in the human body, is still very weak and has become a bottleneck that restricts the further development and utilization of polysaccharides (Han, 2018). The first reason is that polysaccharides with different structures undergo different metabolic elimination in the body, lacking relevant information on their metabolites (Wang K. et al., 2017). On the other hand, there is currently no sensitive, reliable, and standardized detection method, so it is quite difficult to detect the products after polysaccharide metabolism. In recent years, polysaccharide fluorescence labeling and polysaccharide near-infrared fluorescence labeling have been applied to the study of *in vivo* pharmacokinetics of polysaccharides (Wang K. et al., 2017). The pharmacokinetic process of marine polysaccharides is complex and is influenced by various factors, such as the molecular weight, composition, structural characteristics, and mode of administration of polysaccharides. The metabolic transformation process of marine polysaccharides may involve the action of various enzymes, such as glucosidase, phosphatase, and protease, which can decompose polysaccharides and release monosaccharides or oligosaccharides, thereby affecting the bioavailability and efficacy of polysaccharides (Althagbi et al., 2020). Besides, the distribution and metabolism of marine polysaccharides in the body may also be influenced by the body’s immune system and inflammatory responses. Thus, the pharmacokinetic process of marine polysaccharides is a dynamic equilibrium process that is influenced and regulated by multiple factors (Althagbi et al., 2020).

Until now, most of the studies on antiviral effects of marine polysaccharides have been observed *in vitro* or in mouse model systems. Therefore, further studies are needed in order to investigate their antiviral activities in human subjects (Wang et al., 2012). Moreover, the structure–activity relationships and the underlying molecular mechanisms of antiviral actions of marine polysaccharides need to be understood precisely and elucidated clearly by intensive studies in the future (Altmann, 2017). Although, similarly to other charged compounds, marine sulfated polysaccharides may hardly cross the different barriers of the body by oral administration, some studies have showed that vaginal gel therapy of marine polysaccharides had remarkable anti-HSV-2 effects, which suggested that they may be used for prevention and treatment of genital herpes by vaginal administration in the future (Yan et al., 2023). In addition, marine natural product pharmaceuticals still have limitations in terms of resource acquisition, extraction and separation, and efficacy and safety issues (Lu et al., 2021). Thus, developing dosage forms suitable for the administration of marine drugs, especially the marine polysaccharide drugs, will contribute to the further development of marine derived antiviral drugs.

## 7 Outlook and conclusion

Marine derived bioactive compounds especially the algae polysaccharides can effectively block the infection and replication processes of different viruses including HSV, influenza virus and SARS-CoV (**Table 2**) (Levendosky et al., 2015). Besides, more than 70% of the world’s surface is covered by the ocean, which is filled with marine organisms containing various bioactive compounds. Thus, the natural products derived from marine organisms are excellent sources for development of novel anti-HSV agents. In addition, some marine compounds may not only inhibit HSV infection but also enhance the antiviral immune system to accelerate HSV clearance, suggesting that these compounds may be used to treat both primary infection and latent infection of HSV. However, marine nature products especially algae polysaccharides are structurally diverse and heterogeneous, which make the studies of their precise structures challenging, and may also hinder their development as antiviral drugs to date (Witvrouw and De Clercq, 1997). Therefore, the precise structure–activity relationships and pharmacokinetics of marine compounds need to be understood clearly by further studies in the future.

**Table 2:** Different modes of actions of algal polysaccharides against HSV and other viruses.

Algal Polysaccharides	Virus Type	The Stages Affected by Compounds	References
Carrageenan	HSV	Adsorption and replication	(González et al., 1987; Carlucci et al., 1999b)
	Influenza virus	Adsorption and entry	(Leibbrandt et al., 2010)
	Dengue virus	Adsorption and uncoating	(Talarico and Damonte, 2007)
Fucoidan	HSV	Adsorption and entry	(Baba et al., 1988; Mandal et al., 2007; Alboofetileh et al., 2019)
	Influenza virus	Entry and release	(Wang W. et al., 2017)
	SARS-CoV-2	Adsorption and replication	(Oliyaei et al., 2022)
Alginate	HSV	Entry	(Sinha et al., 2010)
	HIV	Replication	(Queiroz et al., 2008)
	SARS-CoV-2	Adsorption and Entry	(Serrano-Aroca et al., 2021)
Ulvan	HSV	Replication	(Tziveleka et al., 2019)
	EV71	Adsorption and replication	(Wang et al., 2018)
	VSV	Replication	(Chi et al., 2020)

In conclusion, marine derived natural compounds, especially the algae polysaccharides, have many advantages, such as relatively low production costs, low cytotoxicity, and wide acceptability, suggesting that marine compounds merit further investigation as promising anti-HSV agents to treat HSV infection related diseases. However, more studies of these anti-HSV lead compounds against clinical strains especially the acyclovir-resistant strains will be required to advance them for drug development. Nevertheless, the marine derived compounds have great potential to be developed into novel anti-HSV candidates for therapy of herpetic encephalitis and genital herpes in the future.

## Author contributions

CH: Funding acquisition, Writing – review & editing, Conceptualization. ZX: Writing – original draft. CX: Writing – original draft. RY: Writing – review & editing.
